# Effects of social assistance on self-rated health

**DOI:** 10.3389/fpubh.2022.918323

**Published:** 2022-10-20

**Authors:** Siqi Shao, Tiantian Che, Deshui Zhou

**Affiliations:** ^1^Liaoning Police College, Department of Public Security Management, Dalian, China; ^2^Dongbei University of Finance and Economics, Dalian, China; ^3^Institute of Finance and Public Management, Anhui University of Finance & Economics, Bengbu, China

**Keywords:** medical assistance, life assistance, self-rated health, anti-poverty, urban residents

## Abstract

Based on the China Health and Retirement Longitudinal Study (CHARLS) data in 2018, medical assistance and life assistance have significant negative influences on self-rated health, found *via* an empirical analysis based on the Oprobit model. Such negative influences are robust based on the substitution of explained variables and propensity score matching. It can be found from a heterogeneity analysis that the negative influences of medical assistance on self-rated health are more significant in urban residents and residents in Central China and East China. Meanwhile, negative influences of life assistance on self-rated health are more significant in urban residents, and residents in Central China, East China, and Northeast China. This study provides empirical evidence to improve the health of residents by using medical assistance and life assistance accurately and offers important policy enlightenments to formulate appropriate social assistance policies.

## Introduction

Residents' health has important significance for economic development. Declined health levels may affect the income level of residents and living quality. Xi Jinping (1), the General Secretary, pointed out that “there will be no overall well-off without health of people.” This fully reflects the positive role of residents' health in anti-poverty and economic development. The fifth Plenary Session of the 19th CPC Central Committee noted that the overall planning of COVID-19 control and social and economic development has to put people's security of life and physical health as a priority and realize the strategy of “health China (2).” People's health is the development goal to realize a moderately prosperous society. At present, China has overcome poverty completely and achieved significant success in the solidification of anti-poverty. However, phenomena of “poverty for illness” and “returning poverty for illness” occur frequently. Health conditions influence labor capacity and make the whole family poor. Poverty alleviation and eradication are protracted wars. Improving residents' health has become the focus of anti-poverty to prevent “poverty due to illness” and “returning to poverty due to illness” caused by residents' health problems. The residents' health includes not only objective physical health but also psychological and social health. The self-evaluation of health in this paper is the subjective expectation and comprehensive evaluation of the resident's health status, not only the evaluation of the actual health status (3). Medical and life assistance are important means of anti-poverty. The influences of medical and life assistance on residents' health during implementation has become an important problem that has to be studied in the academic circle.

## Theoretical basis and research hypotheses

Shahidi et al. (4) studied the impact of social assistance programs in high-income countries on health through a literature search. He concluded that social assistance programs in high-income countries failed to maintain the health of economically vulnerable groups, and the scope and supply degree of existing programs were insufficient to offset the negative impact of severe socio-economic disadvantages on health. It can be seen that social assistance is attracting increasing social attention as an important factor influencing residents' health. As an important social policy of anti-poverty, social assistance will influence residents' health significantly during implementation. However, there are insufficient studies concerning the influences of medical assistance and life assistance on residents' health. For this reason, the effects of medical assistance and life assistance on residents' health will be analyzed *via* an empirical study. Based on the empirical study results, some suggestions were proposed under the premise of theoretical studies and literature views of anti-poverty in Chinese. Besides, medical assistance and life assistance were income supports in cash to individuals or families in this study. At present, international research on social assistance focuses on the combination of theory and reality. Concerning the transformation of assistance services, Yuka Shirase (5), a Japanese scholar, pointed out that the Japanese government determined the goal of the “life protection system^1^” as “independence” to transfer the poor from social exclusion into social return by implementing a series of aid projects. Moreover, financial aid is changing toward diversified forms such as employment independence, daily life independence, and social relation independence. It aims to realize the goals of “better teaching a man to fish than to give him fish,” expanding independence support consulting services, and theoretically improving the importance of social inclusiveness. Concerning medical assistance, Wang Chaoqun and Nelson demonstrated that medical assistance and life assistance could improve the poor individuals' or families' ability to purchase medical services or daily necessities by increasing their income, thus improving their quality of life. Bierman et al. (6) and Liu Jiankun et al. (7) proved that supplementing the income of residents and improving their quality of life is beneficial to increase the health level of residents. HyunSoo et al. (8) carried out an empirical study based on several countries and several aged people during COVID-19 and concluded that relatively high income was positively related to good subjective health. Moreover, the income-health gradient was stronger in countries that had more deaths for COVID-19 but few infection cases. Therefore, it could be predicted from the influencing mechanisms of medical assistance and life assistance on residents' health that both medical assistance and life assistance have significantly positive effects on residents' health. Concerning medical assistance and influences of social relations, Stopka et al. (9), Woolf et al. (10), and Braveman et al. (11) also concluded that public health pays increasing attention to the social determinants of health. Through verification, they found that socioeconomic factors, including individual income, wealth, and education, are the root causes of various health outcomes. However, most of these are socioeconomic factors that influence health conditions. By investigating African American males with low incomes, Mullany et al. (12) concluded that studies on public health must continue to oppose health promotion acts emphasized on individual behaviors. Moreover, health issues not just discuss factors of individual behaviors but must be investigated by long-term histories, politics, and economics.

Changes in social security policies are an important factor influencing residents' health (13, 14), Social assistance is an important social security policy. At present, there are many studies concerning the influences of social assistance on residents' health. Sod-Erdene et al. (15) compared health changes of people with and without social assistance in Ontario, Canada, the UK, and the USA and found that people with social assistance had poorer health conditions or greater differences in health conditions of people without social assistance. Nevertheless, most scholars found that the implementation of social assistance would cause negative influences on residents' health. Wu et al. (16) and Berg et al. (17) found that receiving social welfare in childhood in the United States will lead to depression in adulthood and harm mental health. Qi et al. (18) found that China's lowest social security system exerted negative influences on the psychological health and well-being of recipients. Butterworth et al. (19) and Butterworth (20) found that residents in Australia who received government revenue support had poorer spiritual and psychological health levels than people who did not receive government revenue support. Baigi et al. (21) also found that most people receiving social assistance had sleep disorders, anxiety, physical pains, and a lack of movement. All of these are important influencing factors of health. Therefore, the health level of residents with social assistance is obviously lower than that of residents without social assistance. Medical assistance and life assistance are important components of social assistance. The effects of medical assistance and social assistance on residents' health are consistent. Based on the above empirical study results about the negative influences of social assistance on residents' health, the following hypotheses are proposed.

H1: Receiving medical assistance has significantly negative influences on residents' health.
H2: Receiving life assistance has significantly negative influences on residents' health.

## Empirical method and data

### Data source and selection of variables

Data from the China Health and Retirement Longitudinal Study (CHARLS) used in this study came from the National Development Research Institute of Peking University. All data were nationwide microscopic data of families and individuals over 45 years old in 2015 and 2018, and the interviewees are from 150 county units throughout the country. Basic information, health conditions, and respondents' income were matched and integrated to improve the data quality. After eliminating missing values, 38,570 effective observation values were acquired, conforming to the sample size requirements. All 28 provinces, excluding Tibetan, have extensive representativeness and can effectively reflect self-rated health influences on anti-poverty. Major variables used in this study were introduced as follows:

*Explained variable:* Self-rated health was used as the explained variable, which came from the question in the questionnaire of “What do you think about your health conditions.” The choices of self-rated health status are as follows: (1) Very good; (2) Good; (3) General; (4) Not good; (5) Very bad. In the empirical study, three category variables are generated. The answers “not good” and “very bad” are assigned 0, the “general” is assigned 1, and the “good” and “very good” are assigned 2. *Explanatory variables:* Core explanatory variables include medical assistance and life assistance. Medical assistance and life assistance are important means of social assistance, and their important roles in anti-poverty should be developed fully. Medical assistance refers to a certain amount of cash assistance and medical insurance for urban and rural residents suffering from major diseases and causing medical and family difficulties. It is mainly to play a supporting role beyond basic medical insurance. Specifically, medical assistance came from the question of “Have you received medical assistance in the past year.” In the empirical study, the categorical variable is generated. The answer “no” is assigned 0, and “yes” is assigned 1. Life assistance includes subsidies granted by the government, such as the minimum living allowances, subsidies for five guarantee households, and subsidies for poor households. It is one of the important contents of social assistance and the last line of defense for people living in difficulties. Life assistance came from the question of “Have you received other government subsidies to individuals in the past 1 year?.” In the empirical study, a categorical variable is generated. The answer “no” is assigned 0, and the “yes” is assigned 1.

*Control variables:* To reduce the possible bias caused by missing variables and the possible bias in the statistical model caused by ignoring variables, the following five types of variables are controlled in the empirical analysis: personal characteristics include age, gender, married, registered residence, education level, and retired. Family characteristics include annual family income, number of children, and living with children. Health behaviors and medical characteristics include smoking, drinking, chronic diseases, and self-treatment. Social insurance features include whether to participate in medical insurance and whether to participate in endowment insurance. The regional characteristics are divided into West China, Central China, East China, and Northeast China. Specific definitions are shown in Table 1.

**Table 1:** Variable definitions and descriptive statistics.

**Variables**	**Definition**	**Mean**	**Standard error**
**Self-rated health**	**Dummy variable**		
Bad	Bad = 1, otherwise = 0	0.258	0.437
Fair	Fair = 1, otherwise = 0	0.495	0.500
Good	Good = 1, otherwise = 0	0.247	0.431
Medical assistance	Categorical variable, Yes = 1 and No = 0	0.003	0.056
Life assistance	Categorical variable, Yes = 1 and No = 0	0.041	0.198
Age	Continuous variable	60.800	9.959
Gender	Categorical variable, Male = 1 and Female = 0	0.525	0.499
Married	Categorical variable, Yes = 1 and No = 0	0.809	0.393
Registered residence	Categorical variable, Urban = 1 and Rural = 0	0.394	0.489
**Education leval**	**Dummy variable**		
Primary school and below	Primary school and below = 1, otherwise = 0	0.642	0.480
Middle school	Central school = 1, otherwise = 0	0.229	0.420
Senior high school and technical secondary school	Senior high school and technical secondary school = 1, otherwise = 0	0.105	0.307
College degree, bachelor degree or above	College degree, bachelor degree or above = 1, otherwise = 0	0.024	0.153
Retired	Categorical variable, Yes = 1 and No = 0	0.132	0.339
Annual family income	Continuous variable (log)	7.616	3.731
Number of children	Continuous variable	2.894	1.688
Living with children	Categorical variable, Yes = 1 and No = 0	0.232	0.422
Smoking	Categorical variable, Yes = 1 and No = 0	0.271	0.445
Drinking	Categorical variable, Yes = 1 and No = 0	0.260	0.439
Chronic diseases	Continuous variable	0.714	1.072
Self-treatment	Categorical variable, Yes = 1 and No = 0	0.568	0.495
Participate in medical insurance	Categorical variable, Yes = 1 and No = 0	0.971	0.167
Participate in endowment insurance	Categorical variable, Yes = 1 and No = 0	0.790	0.408
**Regional**	**Dummy variable**		
West China	West China = 1, otherwise = 0	0.331	0.471
Central China	Central China = 1, otherwise = 0	0.287	0.452
East China	East China = 1, otherwise = 0	0.313	0.464
Northeast China	Northeast China = 1, otherwise = 0	0.070	0.255

### Measurement model

The explained variable in this study is the self-rated health of residents. Since the explained variable is the ordered discrete variable of the self-rated health of residents, which is assigned as an integer, an Ordered-probit (“Oprobit”) regression model was established to study the influences of medical assistance and life assistance on the self-rated health of residents. Then, the measurement model can be set as follows:

First, the standard measurement model of influences of medical assistance on self-rated health of residents is as follows:


(1) $$\begin{array}{l} Health_{i}=\alpha_{0}+\alpha_{1}Maid_{i}+\alpha_{2}X_{i}+Year+Provice+\varepsilon_{i} \end{array}$$

(2) $$\begin{array}{l} Health_{i}=\beta_{0}+\beta_{1}Laid_{i}+\beta_{2}X_{i}+Year+Provice+\mu_{i} \end{array}$$

where *Health*_*i*_ refers to the self-rated health of residents. *Maid*_*i*_ and *Laid*_*i*_ are the explanatory variables: medical assistance and life assistance. *X*_*i*_ refers to a series of control variables of the explained variable, including personal characteristics, family characteristics, health behaviors, medical characteristics, social insurance features, and regional characteristics. Further, α_0_, α_1_, α_2_, β_0_, β_1_ and β_2_ are parameters for estimation. The year and province represent the time and province dummy variables. Finally, ε_*i*_ and μ_*i*_ represent residual errors.

Second, this study concerns the relations of medical assistance and life assistance with self-rated health. Since residents receiving medical assistance and life assistance are not decided randomly, residents receiving medical assistance and life assistance have good self-rated health due to an improvement in quality of life. Meanwhile, residents without medical assistance and life assistance had poor self-rated health. Therefore, people can choose whether to accept medical assistance and life assistance or not. Besides, the individual characteristics of residents may influence medical assistance, life assistance, and self-rated health at the same time, resulting in the poor self-selection problem and causing selection bias. The consistent, clean average treatment effect (ATT) of medical assistance and life assistance on self-rated health was estimated by propensity score matching (PSM) to solve the self-choice problem caused by selection bias. The specific model is introduced as follows:


(3) $$\begin{array}{l} ATT=E\left(Y_{1i}|I_{i}=1\right)-E\left(Y_{0i}|I_{i}=1\right) \end{array}$$

where *Y*_1*i*_ refers to the self-rated health condition of residents with medical assistance and life assistance. *Y*_0*i*_ refers to the self-rated health condition of residents without medical assistance and life assistance in the control group when they accept medical assistance and life assistance. Since the practical value of the control group cannot be observed, it is necessary to estimate the value of the control group by establishing a counterfactual framework. Finally, the clean, consistent ATT was gained.

## Standard empirical results

### Comprehensive evaluation of influences of medical assistance and life assistance on self-rated health

Columns (1)–(6) of Table 2 investigated the effects of self-rated health on medical assistance and life assistance. Column (1) and column (4) show that after controlling the fixed time effect and provincial effect, the implementation of medical assistance and life assistance significantly negatively influences self-rated health. After controlling for personal characteristics, family characteristics, health behavior, medical characteristics, social insurance characteristics, and regional characteristics, the regression results in columns (2) and (5) show that the self-rated health level of residents has decreased by 57.0 and 23.5%, respectively, significant at the statistical level of 1%. The regression results in columns (3) and (6) using ologit are still stable. This is consistent with the research hypotheses in this study and the research conclusions of Qi et al. (18) and Shahidi et al. (22).

**Table 2:** Standard regression results.

**Variable**	**Self–rated health**					
	**(1)**	**(2)**	**(3)**	**(4)**	**(5)**	**(6)**
Medical assistance	−0.681^***^ (0.120)	−0.570^***^ (0.124)	−1.021^***^ (0.209)			
Life assistance				−0.304^***^ (0.030)	−0.235^***^ (0.031)	−0.407^***^ (0.053)
Age		−0.011^***^ (0.001)	−0.018^***^ (0.001)		−0.010^***^ (0.001)	−0.018^***^ (0.001)
Gender		−0.010 (0.012)	−0.017 (0.020)		−0.009 (0.012)	−0.015 (0.020)
Married		0.041^**^ (0.015)	0.068^**^ (0.010)		0.037^**^ (0.015)	0.061^**^ (0.027)
Registered residence		0.148^***^ (0.014)	0.252^***^ (0.023)		0.149^***^ (0.014)	0.252^***^ (0.023)
**Education level** **(Reference: primary school and lower)**						
Middle school		0.141^***^ (0.015)	0.243^***^ (0.025)		0.140^***^ (0.015)	0.242^***^ (0.025)
Senior high school and technical secondary school		0.275^***^ (0.020)	0.464^***^ (0.033)		0.273^***^ (0.020)	0.459^***^ (0.033)
College degree, bachelor degree or above		0.452^***^ (0.038)	0.745^***^ (0.062)		0.447^***^ (0.038)	0.739^***^ (0.062)
Retired		0.077^***^ (0.018)	0.128^***^ (0.031)		0.074^***^ (0.018)	0.124^**^8 (0.031)
Annual family income		0.003 (0.002)	0.005^*^ (0.003)		0.003 (0.002)	0.005^*^ (0.003)
Number of children		−0.007^*^ (0.004)	−0.012^**^ (0.006)		−0.007^**^ (0.004)	−0.012^*^ (0.006)
Living with children		0.045^**^ (0.015)	0.075^**^ (0.025)		0.045^**^ (0.015)	0.076^**^ (0.025)
Smoking		0.047^**^ (0.014)	0.081^**^ (0.024)		0.051^***^ (0.014)	0.087^***^ (0.024)
Drinking		0.231^***^ (0.014)	0.390^***^ (0.024)		0.233^***^ (0.014)	0.394^***^ (0.024)
Chronic diseases		−0.273^***^ (0.006)	−0.463^***^ (0.010)		−0.273^***^ (0.006)	−0.463^***^ (0.010)
Self-treatment		−0.024^**^ (0.012)	−0.039^*^ (0.020)		−0.023^*^ (0.012)	−0.038^*^ (0.020)
Participate in medical insurance		−0.048 (0.035)	−0.072 (0.059)		−0.049 (0.035)	−0.074 (0.059)
Participate in endowment insurance		0.004 (0.015)	0.006 (0.025)		0.003 (0.015)	0.005 (0.025)
**Regions** **(References: East China)**						
Central China		0.293^***^ (0.037)	0.513^***^ (0.064)		0.293^***^ (0.037)	0.511^***^ (0.064)
East China		0.484^***^ (0.038)	0.844^***^ (0.065)		0.483^***^ (0.038)	0.840^***^ (0.065)
Northeast China		0.332^***^ (0.043)	0.595^***^ (0.073)		0.326^***^ (0.043)	0.584^***^ (0.074)
Province constant	Control	Control	Control	Control	Control	Control
Time constant	Control	Control	Control	Control	Control	Control
Prob > chi2	0.000	0.000	0.000	0.000	0.000	0.000
Ps–R2	0.011	0.064	0.064	0.012	0.064	0.064
Observations	38,570	38,570	38,570	38,570	38,570	38,570

^***^, ^**^, and ^*^ indicates significance on the statistical levels of 1, 5, and 10%, respectively. The numbers in the brackets are the standard errors of coefficient robustness.

Accepting medical assistance will cause negative influences on the self-rated health of residents. This might be due to poor health consciousness before accepting medical assistance. Residents have poor knowledge of their actual health conditions, so they often blindly overestimate their physical level and have relatively high self-rated health. The implementation of medical assistance can increase the health consciousness of residents effectively. After accepting medical assistance, the health consciousness of residents is enhanced, and they can understand physical conditions clearly. However, medical assistance provides basic medical services, and it cannot improve residents' health effectively, resulting in the low self-rated health level of residents. Therefore, accepting medical assistance has negative influences on residents' self-rated health.

Accepting life assistance has negative influences on the self-rated health of residents. Possible reasons are introduced as follows. On the one hand, the implementation of life assistance can assure the lowest living standard of residents and solves the problem of absolute poverty. After the basic life rights of residents are secured, they will pursue a higher level of health: psychological health. However, residents' needs to be respected when accepting life assistance often cannot be met, which may affect their psychological health. The psychological health of residents is an important component of self-rated health. Decreased psychological health leads to a reduction in self-rated health. On the other hand, life assistance has strict conditions for application, and most residents who can accept life assistance are unemployed. Although life assistance can relieve the anxiety of unemployment to some extent, it still cannot make up for the negative influences on psychological health. At present, some academic studies have proved that unemployment will decline the health level of residents (23–25). Moreover, with the continuous improvement of China's economic development level, the marginal effect brought by life assistance decreases gradually. Therefore, accepting life assistance may have negative influences on the self-rated health of residents.

The estimation coefficient of control variables is also worthy of note. Take column (2), for an example. The estimation coefficient of *age* is significantly negative at the 1% level. This might be because the risks of residents being influenced by diseases and the aged are increased. Meanwhile, the self-rated health level of residents decreases with the increase of age. The estimation coefficient of *gender* is negative but not significant, indicating that the correlation between gender and residents' self-rated health is poor. The estimated coefficient of *married* is significantly positive, which may be because the spouses of married residents can give each other greater support in life and psychology, conducive to improving their health. The estimated coefficient of a *registered residence* is significantly positive, which may be because urban residents have higher health awareness, pay more attention to their health, and have better medical access. This can effectively improve the health of residents. The estimated coefficient of *retired* is significantly positive, which may be because retirees have longer leisure time and can better pay attention to their health and effectively improve their health level. The estimation coefficient of *education* is significantly positive at the 1% level; with the increase of the education level, the estimation coefficient increases. This indicates that the self-rated health level of residents is positively related to the education level. This also reflects the return on investment to education to some extent. The estimation coefficient of *medical insurance* is significantly negative. Since basic medical insurance for urban and rural residents in China is compulsory, residents with supplementary medical insurance have poor self-rated health, potentially caused by adverse selection. Since residents relatively understand their physical conditions, residents with poor health conditions will participate in supplementary medical insurance by using information asymmetry to avoid poverty caused by diseases. Residents with good self-rated health may not participate in supplementary medical insurance. As a result, residents with supplementary medical insurance have poor self-rated health conditions.

The estimated coefficient of the *annual family income* is positive but not significant in the family characteristics. The estimated coefficient of *the number of children* is significantly negative. The reason may be that the increase in the number of children will disperse the medical resources available to family members to a certain extent, which is not conducive to the health improvement of residents. The estimated coefficient of *living with children* is significantly positive, which may be because living with children can obtain not only economic support but also get psychological comfort and can effectively improve their health level. The estimated coefficient of *smoking* and *drinking* in health behavior and medical characteristics is significantly positive. The possible reason is that residents who smoke and drink overestimate their health levels. The estimated coefficient of *chronic disease* and *self-treatment* is significantly negative, which may be because the physical quality of the residents with chronic disease and self-treatment is poor and will not improve in a short time. The estimated coefficient of *participating in medical insurance* is negative. Still, it is not significant, which is related to adverse selection in the participation process, and the insured's health status is poor. The estimation coefficient of *participating in the endowment insurance* is positive but not significant. In the regional characteristics, compared with *West China*, the estimated coefficients of *Central China, East China*, and *Northeast China* are significantly positive, and the estimated coefficients show that *East China* > *Northeast China* > *Central China*. The possible reason is that the closer to *East China*, the stronger the residents' health awareness, and the better the effectiveness and accessibility of medical services, which is consistent with reality.

### Robustness test

#### Substitution of dependent variables

This paper replaces the explanatory variables to test the robustness of the results and the robustness of the above regression results. According to the design of the Charles questionnaire, we selected the following question “how likely are you to live to this age? This question reflects the expectation of your own health.” The answers of the respondents were generated into three categories of variables. The answers “almost impossible” and “unlikely” were assigned 0, the “possible” was assigned 1, and the “likely” and “almost certain” were assigned 2. Generally speaking, the longer the life expectancy, the better the evaluation of one's health. Table 3 shows the regression results of alternative variables. The results show that the life expectancy of residents receiving medical assistance and living assistance is shorter, consistent with the regression results in Table 2, further proving the robustness of the above results.

**Table 3:** Robustness test — substitution of the explained variables.

	**Life expectancy evaluation**	
Medical assistance	−0.375^***^ (0.119)	
Life assistance		−0.068^***^ (0.034)
Control variables	Control	Control
Province constant	Control	Control
Province constant	Control	Control
Prob > chi2	0.000	0.000
Ps–R2	0.52	0.052
Observations	31,046	31,046

*** indicates significant on the statistical level of 1%, respectively. Numbers in the brackets are standard errors of coefficient robustness.

#### Processed the self-selection problem

PSM was applied to estimate the net influences of internet uses on medical expenses, which could relieve bias caused by self-selection to some extent. First, it is estimated through the latest matching. Second, it is verified by radius matching and kernel matching. The premise of PSM is that the equilibrium of samples is verified. It can be seen from the Table 4 that the standard deviations of samples after the nearest neighbor matching have all decreased to lower than 10%. The standard deviation of variables is decreased significantly. In other words, the characteristic differences of samples are controlled to some extent. The results of PSM have good explanatory power. Besides, it was found from the balance test that PSM affects solving residents' self-choice problems.

**Table 4:** Sample balance test of propensity score matching.

**Variable**	**Sample**	**Processing**	**Control**	**Standard**	**Deviation**	***T*-value**	***P*-value**
		**group**	**group**	**deviation% %**	**reduction %**		
Age	Before	62.697	60.794	19.6		2.11	0.035
	After	62.697	63.133	−4.5	77.1	−0.34	0.734
Gender	Before	0.615	0.525	18.2		1.99	0.047
	After	0.615	0.590	5.0	72.7	0.39	0.696
Married	Before	0.770	0.810	−9.6		−1.10	0.273
	After	0.770	0.773	−0.5	94.8	−0.04	0.970
Registered residence	Before	0.254	0.395	−30.3		−3.17	0.002
	After	0.254	0.281	−5.7	81.0	−0.47	0.640
Education	Before	0.508	0.511	−0.4		−0.04	0.967
	After	0.508	0.504	0.5	−42.1	−0.04	0.967
Retired	Before	0.123	0.132	−2.8		−0.31	0.760
	After	0.123	0.121	0.6	78.2	0.05	0.961
Annual family income	Before	7.976	7.615	10.5		1.07	0.286
	After	7.976	7.668	8.9	14.8	0.71	0.478
Number of children	Before	3.320	2.893	23.4		2.79	0.005
	After	3.320	3.283	2.0	91.4	0.15	0.885
Living with children	Before	0.213	0.233	−4.7		−0.51	0.612
	After	0.213	0.236	−5.4	−16.1	−0.42	0.675
Smoking	Before	0.221	0.271	−11.6		−1.24	0.215
	After	0.221	0.195	6.2	46.7	0.51	0.610
Drinking	Before	0.164	0.260	−23.7		−2.42	0.015
	After	0.164	0.152	3.0	87.2	0.26	0.793
Chronic diseases	Before	1.156	0.713	36.8		4.55	0.000
	After	1.156	1.250	−7.8	78.7	−0.52	0.606
Self–treatment	Before	0.639	0.568	14.5		1.58	0.114
	After	0.639	0.664	−5.0	65.4	−0.40	0.688
Participate in medical insurance	Before	0.959	0.971	−6.7		−0.81	0.418
	After	0.959	0.947	6.7	−0.2	0.45	0.652
Participate in endowment insurance	Before	0.770	0.790	−4.6		−0.52	0.603
	After	0.770	0.768	0.5	89.3	0.04	0.970
Regions	Before	0.836	1.122	−30.7		−3.31	0.001
	After	0.836	0.848	−1.3	95.7	−0.10	0.918

Before matching, PS R2, LR chi2, and the mean and median of the standard deviation were 0.041, 67.48, 15.4, and 13.1, respectively; After matching, the corresponding values are 0.008, 2.83, 4.2, and 5.0, so the matching effect is good.

The ATT of the self-rated health of residents who receive medical assistance and life assistance is shown in Table 5. When other variables are controlled, the self-rated health of the control group and user group that accepts medical assistance and life assistance and their differences were estimated. According to the nearest neighbor matching results, ATT values of residents' self-rated health before matching were −0.350 and −0.152, which were changed to −0.450 and −0.196 after the matching. This implies that the processing group and control group accepting medical assistance and life assistance have different influences on residents' self-rated health. This is difficult to observe in an ordinary regression analysis. In this study, calipers matching and Kernel matching were further used. Results are consistent with the nearest neighbor matching. Therefore, the conclusion that accepting medical assistance and life assistance has significantly negative influences on the self-rated health of residents is robust.

**Table 5:** Self-choice processing of residents with medical assistance and life assistance.

		**Categorical**	**Processing group**	**Control group**	**ATT value**	**Standard deviatio**	***T*-value**
Medical assistance	Nearest neighbor matching	Before	0.541	0.991	−0.450	0.064	−6.98^***^
		After	0.541	0.891	−0.350	0.070	−4.99^***^
	Radius matching	Before	0.541	0.991	−0.450	0.064	−6.98^***^
		After	0.541	0.958	−0.417	0.063	−6.62^***^
	Kernel matching	Before	0.541	0.991	−0.450	0.064	−6.98^***^
		After	0.541	0.986	−0.445	0.063	−7.08^***^
Life assistance	Nearest neighbor matching	Before	0.802	0.997	−0.196	0.018	−10.71^***^
		After	0.802	0.953	−0.152	0.020	−7.47^***^
	Radius matching	Before	0.802	0.997	−0.196	0.018	−10.71^***^
		After	0.802	0.939	−0.137	0.019	−7.34^***^
	Kernel matching	Before	0.802	0.997	−0.196	0.018	−10.71^***^
		After	0.802	0.973	−0.171	0.019	−9.22^***^

The nearest neighbor matching adopts one-to-four matching. The radius in the radius matching is 0.01, and the Markov matching chooses the default value. The default values are used for kernel function and bandwidth in kernel matching.

*** indicates significance on the statistical levels of 1%.

## Heterogeneity analysis

Medical and life assistance have different impact effects on different residents. To understand the impact effects on different groups, the following heterogeneity analysis is made:

When the explained variable is medical assistance, the heterogeneity analyses under different division standards are introduced as follows.

Frist, Model 1 in Table 6 divides residents into different groups according to household registrations to analyze the influences of medical assistance on the self-rated health of urban and rural residents. According to the regression results, medical assistance has significant negative influences on the self-rated health of both urban and rural residents. Such influences are significant at the statistical levels of 5%. This is consistent with the previous basic regression results. It can be found from the comparison of coefficients that the negative influences of medical assistance on the self-rated health of urban residents are stronger than those of rural residents. This can be explained as follows. With the high-quality economic development, the urban-rural income gap is narrowing continuously, and people's consciousness of health is improving continuously. Both urban and rural residents' understanding of their health conditions is improved greatly. There is good accessibility to medical services in urban areas, but medical service prices in rural areas are lower. Since medical assistance provides basic medical series to residents, cash support from medical assistance can purchase more medical services in rural areas, improving the health levels of rural residents greatly. Relatively speaking, urban residents can only buy limited medical services with medical assistance, resulting in limited promotion on the health level of residents. Hence, it has to determine medical assistance treatment according to different development levels in urban and rural regions. Therefore, the negative influences of medical assistance on the self-rated health of urban residents are stronger than those of rural residents.

**Table 6:** Heterogeneity analysis under different division standards.

	**Division standards**		**Self-rated health**	**Control variables**	**Province and time constant**	**R-squared**	**Observations**
Medical assistance	Household registration	Rural	−0.333^**^ (0.126)	Control	Control	0.061	23,369
	(Model 1)	Urban	−1.149^**^ (0.224)	Control	Control	0.063	15,201
	Region	West China	−0.170 (0.156)	Control	Control	0.054	12,768
	(Model 2)	Central China	−0.949^***^ (0.223)	Control	Control	0.064	11,051
		East China	−0.962^***^ (0.237)	Control	Control	0.062	12,063
		Northeast China	−0.721 (0.533)	Control	Control	0.062	2,688
Life assistance	Household registration	Rural	−0.211^***^ (0.037)	Control	Control	0.061	23,369
	(Model 3)	Urban	−0.503^***^ (0.126)	Control	Control	0.037	15,201
	Region	West China	−0.125^**^ (0.049)	Control	Control	0.051	12,768
	(Model 4)	Central China	−0.311^***^ (0.053)	Control	Control	0.062	11,051
		East China	−0.257^***^ (0.056)	Control	Control	0.057	12,063
		Northeast China	−0.300^**^ (0.133)	Control	Control	0.063	2,688

^***^ and ^**^ indicates significance on the statistical levels of 1 and 5%.

Second, Model 2 divides residents according to regional characteristics to analyze the influences of medical assistance on residents' self-rated health in different regions. Medical assistance has significant negative influences on residents' self-rated health in East China and Central China, significant at the statistical levels of 1%. Medical assistance also has negative influences on residents' self-rated health in *West China* and *Northeast China*, but such influences are not significant. This is basically consistent with basic regression results in the above text. Since the estimation coefficient of *West China* and *Northeast China* is not significant, the estimation coefficients of *East China* and *Central China* were compared. Compared with East China, medical assistance has stronger negative influences on the self-rated health of residents in Central China. This might be because residents in Central China have poor health consciousness and pay less attention to their health conditions than those in East China. They are more likely to overestimate their health level. However, medical assistance only provides basic medical services. In fact, medical assistance provides little financial aid, insufficient to purchase enough medical services to improve the health level greatly. Consequently, the self-rated health level of residents in Central China is relatively low.

When the explanatory variable is life assistance, the heterogeneity analyses under different division standards are introduced as follows.

Frist, Model 3 divides residents into different groups according to household registration to analyze the influences of life assistance on the self-rated health of urban and rural residents. According to the regression results, life assistance has significant negative influences on the self-rated health of both urban and rural residents. Such influences are significant at the statistical levels of 1%. This is consistent with the previous basic regression results. It can be found from a comparison of the coefficients that the negative influences of life assistance on the self-rated health of urban residents are stronger than those of rural residents. Compared with rural residents, urban residents have a stronger desire to pursue higher needs. When life assistance meets the basic living needs of urban residents, they begin to pursue the higher level of needs. However, life assistance cannot meet such needs of urban residents, affecting their psychological health. Psychological health is an important part of residents' self-rated health, and self-rated health declines if a higher level of needs cannot be met. Moreover, the urban economic level is higher than in rural areas. The marginal effect brought by life assistance is smaller than in rural areas.

Third, Model 4 divides residents according to regional characteristics to analyze the influences of life assistance on residents' self-rated health in different regions. The impact of living assistance on residents' self-rated health in different regions is significantly negative, at least at the statistical level of 5%. Compared with *West China*, the negative impact of living assistance on Residents' health is stronger in Central China, East China, and Northeast China. The living assistance is mainly to meet the basic living needs of residents. The economic development level of Central China, East China, and Northeast China is higher, and the income level of residents is higher. However, the income of residents in *West China* is relatively low, so the marginal effect of living assistance is in West China > Central China, East China, and Northeast China. Therefore, compared with *West China*, the negative effect of residents receiving life assistance on self-rated health is stronger in *Central China*, East China, and Northeast China.

## Research conclusion and prospect

### Research conclusion

Based on CHARLS data in 2018, the influences of medical assistance and life assistance on self-rated health were investigated by the Oprobit model. First, medical assistance and life assistance have significant negative influences on residents' self-rated health. Self-rated health is also influenced by external factors, such as age, gender, household registration, education level, and regions. Second, the basic regression results' robustness was tested by substituting explained variables and PSM. Results are consistent with the basic regression results. Finally, medical assistance and life assistance influences on self-rated health were analyzed from household registration and region. Results demonstrate that the negative influences of medical assistance on self-rated health are more significant in urban residents and residents in East China and Central China. The negative influences of life assistance on self-rated health are more significant in urban residents and residents in Central China, East China, and Northeast China.

In 2020, absolute poverty was eliminated in China, and China entered into a moderately prosperous society. Currently, China has entered into the post-poverty era, and overcoming poverty is a protracted war to prevent the return to poverty. Moreover, improving the quality of life and well-being of people and reaching a new level is a priority in poverty elimination in China. In the future, it is suggested to stick to the realistic, accurate poverty alleviation strategy and relieve poverty according to practical conditions. Moreover, the needs of specific groups should be coped with by professional organizations, special systems, and professional personnel. Based on the above analysis, the author proposes the following policy suggestions to develop the effects of medical assistance and life assistance on the health of residents, aiming to provide beneficial references to improve anti-poverty quality.

### Policy suggestion

First, basic medical treatment and public health knowledge should be provided to increase the health consciousness of male residents and urban residents. Given the frequent occurrences of poverty for diseases, the health consciousness of residents becomes increasingly important. Hence, it is necessary to improve the health consciousness of residents and take preventive measures. Second, the scopes of medical assistance and life assistance should be expanded on to include prevention into the aid system. Existing medical assistance and life assistance are mainly provided to residents who return to poverty for diseases. Including prevention in the aid system would be beneficial to strengthen the health consciousness of residents and decrease the risk of poverty for diseases. Third, the assistance form should be diversified. At present, medical assistance and life assistance are mainly provided in cash, ignoring the important role of physical substances and service assistance. Providing residents life support and medical services through physical and service assistance directly can prevent the misuse of medical assistance and life assistance effectively, improving the health level of residents. Fourth, the assistance objectives should be perfected. At present, the survival assistance objective is realized by providing residents with income support to assure their rights of survival. However, the survival assistance objective alone cannot improve the health conditions of residents. It is suggested to include development-oriented assistance into the assistance objectives and provide residents appropriate employment training per the ability evaluation, improving their income level and health conditions.

### Research limitations and prospects

Since the questionnaire design of the CHARLS database used in the study is not aimed at the research questions, there are certain errors in the data matching and calculation, resulting in certain limitations in the model construction and operation. Future research can consider obtaining data in different ways to improve the explanatory power of the conclusions to the reality. In addition, based on this study, we can conduct research on the implementation effect of social assistance policies in different countries in the future to further verify the relationship between medical assistance and life assistance and residents' health.

## Data availability statement

The raw data supporting the conclusions of this article will be made available by the authors, without undue reservation.

## Author contributions

SS and DZ designed the study and conducted the primary statistical analysis. SS and TC contributed to the writing. All authors contributed to the revisions. All authors contributed to the article and approved the submitted version.

## Conflict of interest

The authors declare that the research was conducted in the absence of any commercial or financial relationships that could be construed as a potential conflict of interest.

## Publisher's note

All claims expressed in this article are solely those of the authors and do not necessarily represent those of their affiliated organizations, or those of the publisher, the editors and the reviewers. Any product that may be evaluated in this article, or claim that may be made by its manufacturer, is not guaranteed or endorsed by the publisher.

## Footnotes

^1^The social assistance system is called the life protection system in Japan, which is the system that guarantees the lowest living standard of the poor.
